# A Novel Missense Mutation in the CLPP Gene Causing Perrault Syndrome Type 3 in a Turkish Family

**DOI:** 10.4274/jcrpe.2717

**Published:** 2016-12-01

**Authors:** Fatma Dursun, Hussein Sheikh Ali Mohamoud, Noreen Karim, Muhammad Naeem, Musharraf Jelani, Heves Kırmızıbekmez

**Affiliations:** 1 Ümraniye Training and Research Hospital, Clinic of Pediatric Endocrinology, İstanbul, Turkey; 2 King Abdulaziz University, Princess Al-Jawhara Albrahim Centre of Excellence in Research of Hereditary Disorders, Jeddah, Saudi Arabia; 3 St. George’s University of London, Human Genetics Research Centre, Division of Biomedical Sciences, London, United Kingdom; 4 Quaid-I-Azam University Faculty of Biological Sciences, Medical Genetics Research Laboratory, Department of Biotechnology, Islamabad, Pakistan; 5 Khyber Medical University, Institute of Basic Medical Sciences, Department of Biochemistry, Medical Genetics and Molecular Biology Unit, Peshawar, Pakistan

**Keywords:** Secondary amenorrhea, Perrault syndrome, CLPP

## Abstract

Perrault syndrome (PRLTS) is a heterogeneous group of clinical and genetic disorders characterized by sensory neuronal hearing loss in both sexes and premature ovarian failure or infertility in females. Neurological and hearing loss symptoms appear early in life, but female infertility cannot be detected before puberty. Spastic limbs, muscle weakness, delayed puberty and irregular menstrual cycles have also been observed in PRLTS patients. Mutations in five genes, i.e. HSD17B4, HARS2, CLPP, LARS2, and C10orf2, have been reported in five subtypes of PRLTS. Here, we report a milder phenotype of PRLTS in a Turkish family in which two affected patients had no neurological findings. However, both were characterized by sensory neuronal hearing loss and the female sibling had secondary amenorrhea and gonadal dysgenesis. Genome-wide homozygosity mapping using 300K single-nucleotide polymorphism microarray analysis together with iScan platform (Illumina, USA) followed by candidate gene Sanger sequencing with ABI 3500 Genetic Analyzer (Life Technologies, USA) were used for molecular diagnosis. We found a novel missense alteration c.624C>G; p.Ile208Met in exon 5 of the CLPP at chromosome 19p13.3. This study expands the mutation spectrum of CLPP pathogenicity in PRLTS type 3 phenotype.

**WHAT IS ALREADY KNOWN ON THIS TOPIC?**

Mutations in five genes -HSD17B4, HARS2, CLPP, LARS2, and C10orf2- have been reported in five subtypes of Perrault syndrome.

**WHAT THIS STUDY ADDS?**

We found a novel missense alteration c.624C>G; p.Ile208Met in exon 5 of the CLPP at chromosome 19p13.3. This study expands the mutation spectrum of CLPP pathogenicity in Perrault syndrome type 3 phenotype.

## INTRODUCTION

Perrault syndrome (PRLTS) is a rare autosomal recessive disorder leading to pure gonadal dysgenesis in affected females (46,XX) and sensorineural hearing loss (SNHL) or deafness in males. Ovarian dysfunction ranges from absent or streak gonads to primary ovarian insufficiency defined as cessation of menses before age 40 years (1). Central nervous system findings have also been reported with this syndrome. Neurologic features described in some affected women include developmental delay, intellectual disability, cerebellar ataxia, and motor and sensory peripheral neuropathy (1).

Pathogenic alterations in five genes have been reported in five subtypes of PRLTS. PRLTS type 1 is caused by mutations in HSD17B4 gene at chromosome 5q23.1 (2) and PRLTS1 patients may present with hearing loss, ovarian dysgenesis leading to female infertility, male infertility, ataxia, and peripheral neuropathy (2,3,4). PRLTS type 2 is caused by mutations in HARS2 at chromosome 5q31.3 and is characterized by deafness in both males and females and gonadal dysgenesis in female patients only (5). PRLTS type 3 is caused by mutations in CLPP gene at chromosome 19p13.3 (6,7). PRLTS3 patients may present with progressive hearing loss, female infertility and premature menopause, microcephaly, epilepsy, growth and mental retardation (6,7). PRLTS type 4 is caused by mutations in LARS2 gene at chromosome 3p21.31and is characterized by hearing loss and premature ovarian failure (8). PRLTS type 5 is caused by mutations in C10orf2 gene at chromosome 10q24.31 (9). PRLTS5 patients may present with progressive ataxia, axonal neuropathy, hyporeflexia, abnormal eye movements, progressive hearing loss, and ovarian dysgenesis (9).

**Figure 1:**
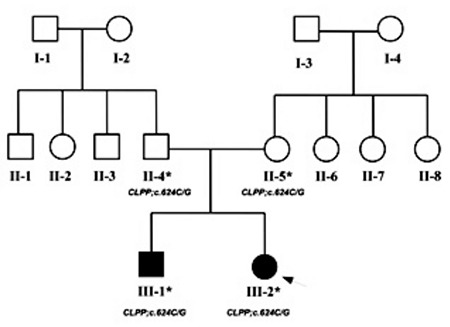
Pedigree of the parents showing autosomal recessive mode of inheritance in the affected individuals. The index patient is indicated with an arrow. The asterisk (*) indicates the samples that were validated by Sanger sequencing with their respective genotypes below each symbol

Here, we report the clinical and molecular investigations of two PRLTS patients from a Turkish family (Figure 1).

## CASE REPORTS

### Patient 1

The patient was a 16-year-old girl (III-2) who presented with secondary amenorrhea. She was attending a special school for hearing-impaired students. The parents were both healthy and non-consanguineous but came from the same village. There were no dysmorphic findings or evidence of other systemic disease in the physical examination. Her weight was 51 kg (25p), height was 160 cm (25-50p), axillary hair was present, pubic hair was at stage 5, and breast development was bilaterally at stage 3 according to the Tanner staging. Neurologic examination was normal. Pelvic ultrasonography revealed a uterus of 8x12x50 mm in size, but ovaries could not be detected. Whole blood count, renal functions, liver functions, as well as glucose and electrolyte levels were within normal ranges, while hormone studies revealed hypergonadotropic hypogonadism. Luteinizing hormone was 20.7 mIU/mL, follicle stimulating hormone was 63.8 mIU/mL, and estradiol was 15 pg/mL. The karyotype was 46,XX. Adrenal steroid levels and thyroid functions were also normal. Hormone replacement treatment with estrogen was initiated. The patient was suspected to have PRLTS because of gonadal failure in association with bilateral sensorineural deafness. Repeated neurologic examination was normal as well as the brain magnetic resonance imaging.

### Patient 2

He was the 21-year-old brother (III-1) of our first patient. He was invited to the clinic because of his hearing loss and a sibling with the clinical diagnosis of PRLTS. He had also attended a school for hearing-impaired students. Physical examination revealed no dysmorphic findings. He was in Tanner stage 5 of puberty with a height and weight of 170 cm [-0.95 standard deviation score (SDS)] and 75 kg (+0.36 SDS), respectively. Neurologic examination was normal. However, he was under the supervision of a psychiatrist and receiving risperidone because of attention-deficit disorder.

### Genetic Analysis

### Homozygosity Mapping

**Figure 2:**
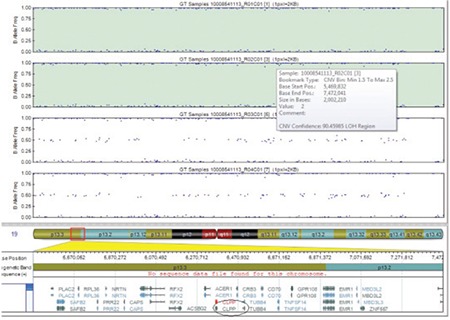
Single-nucleotide polymorphism microarray analysis showing a common region of homozygosity in the affected individuals flanking CLPP gene on chromosome 19p13.3

Genome-wide homozygosity mapping on four family members (unaffected parents and the two affected siblings) was performed using 300K single-nucleotide polymorphism (SNP) microarray (HumanCytoSNP12.2 chip) along with iScan platform (Illumina, USA). We found that a region on 19p13.3 was homozygous in the two affected individuals and was heterozygous in the two parents (Figure 2). This 2 Mb region (chr19:5469832-7472041) contained 64 genes including CLPP according to human genome map (Annotation release 105 http://www.ncbi.nlm.nih.gov/projects/mapview/).

### Sanger Sequencing

**Figure 3:**
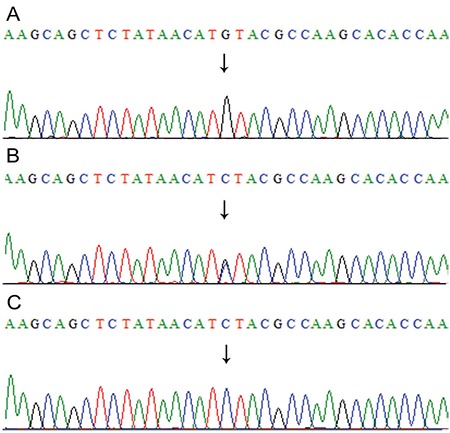
Sequencing analysis of the CLPP gene exon 5 showing C to G transversion at nucleotide position 624 (c.624C>G) in the affected patients (A), heterozygous carrier parents (B), and unaffected siblings or healthy controls (C)

Genomic sequence of the wild-type CLPP gene (ENSG00000125656) was obtained from Ensembl Genome Browser (www.ensembl.org). The six coding exons including exon-intron boundaries were polymerase chain reaction amplified with the primers sets (in Table 1) and sequenced with ABI3500 Genetic Analyzer according the manufacturer’s instructions (Life Technologies, USA). We found a novel homozygous transversion alteration, cytosine to guanine, in exon 5 at nucleotide 624 (c.624C>G) of CLPP gene causing alteration of isoleucine to methionine at 208 amino acid position (p.Ile208Met). Both parents were heterozygous (carriers) for this variant confirming the autosomal recessive inheritance of PRLTS3 phenotype in this family (Figure 3). Sequencing of 100 unaffected healthy individuals (200 chromosomes) excluded the probability of neutral polymorphism of the variant (CLPP; c.624C>G) identified in our patients. Computational prediction software (SIFT, Polyphen-2 and Mutation Taster) declared this alteration as protein damaging. Furthermore, this variant (chr19:6366337C>G) had not been listed in 1000 human genome (http://browser.1000genomes.org/) in 60,706 individuals in the Exome Aggregation Consortium (http://exac.broadinstitute.org/) databases.

## DISCUSSION

Genetic analysis of PRLTS remained unresolved until the first gene was discovered in 2010 (2). Since then, several familial and sporadic cases have been reported, of which, the majority was of European descent (2,5,8). PRLTS3 gene was identified in three Pakistani families (6) and we also screened a family from Saudi Arabia very recently (7). To date, only four mutations including one splicing (c.270+A>G) and three missense (c.433A>C; p.Thr145Pro, c.440G>C; p.Cys147Ser, c.685T>G; p.Tyr229Asp) have been identified in the CLPP gene (6,7). Here, we present, for the first time, a novel CLPP alteration in a Turkish family.

The CLPP enzyme is a 277 amino acid-long peptidase which works in the presence of ATP and magnesium cleaving of larger proteins to smaller peptides (10,11,12). Accumulation of CLPX, mtDNA, and inflammatory factors in tissues have been observed in mice mutants due to CLLP loss of function leading to infertility, hearing loss, and growth retardation (13). A similar mechanism might be involved in humans with PRLTS3 carrying CLPP alterations (6,7).

Molecular diagnosis of PRLTS is efficiently performed through genome-wide SNP microarray for linkage analysis followed by candidate gene sequencing or by directly stepping into whole exome sequencing. These methods can either be utilized individually (2,5,6,8,9) or by combing the two strategies (7). Exome analysis has the advantage of finding causative variants more efficiently compared to candidate gene screening in rare genetic disorders (14,15). However, genome-wide SNP microarray genotyping or array comparative genomic hybridization (CGH) has the advantage of finding out the chromosomal aberrations (16), which may not be possible through whole exome analysis alone. The SNP microarray can also exclude known PRTLS candidates to pin point a single region of homozygosity in ethnically isolated populations (6,17). Here, we found the genome-wide SNP microarray analysis followed by candidate (CLPP) gene sequencing as a successful strategy for identifying the causative variant underlying PRLTS3 in an isolated Turkish family.

In the clinical diagnosis of PRLTS, SNHL and neurological abnormalities both in males and females and female ovarian dysgenesis are considered key findings (1,18). Amenorrhea, gonadal dysgenesis, and SNHL were present in our index patient. However, all these signs may not be detected in younger patients (7). For example, patients with ovarian failure may present with lack of female sexual characteristics, or with primary or secondary amenorrhea. In such cases, pathogenic variants in various causative genes involved in ovarian dysgenesis could be of help in precise diagnosis (19,20). Autoimmunity is also considered as one of the important exclusion factors in patients with ovarian insufficiency, especially in secondary amenorrhea cases (21,22). Congenital disorders of adrenal and gonadal steroidogenesis are also rare causes of ovarian failure (23). Similarly, hearing loss is reported to be present in approximately 50% of women with Turner syndrome (18). For this reason, girls with delayed puberty or amenorrhea with low estrogen and raised gonadotropins need to be investigated either by karyotyping or array CGH analysis to exclude abnormalities of the X chromosome (20,24,25,26).

In addition to SNHL and ovarian insufficiency, neuromuscular abnormalities (spastic diplegia, dysarthria, titubation of the head, hyporeflexia, sensory neuropathy, demyelinating polyneuropathy, cerebellar ataxia, nystagmus, ophthalmoplegia, ptosis, seizures), developmental abnormalities (microcephaly, delayed motor and mental development, learning disabilities), and dysmorphic findings (pes cavus, pes equinovarus, contracted heel cords, atypical facial features, short neck) were found to be associated with PRLTS1 (2,3,27,28,29). These features were not observed in our cases. Previously, we and others reported that short stature, microcephaly, seizures, moderate learning difficulties, and truncal and cerebellar ataxia with signs of lower limb spasticity may occur in PRLTS3 (6,7). Neurologic disabilities, which started by the 18th month and worsened through years, were defined in two siblings with a CLPP mutation in Pakistani and Saudi families (8), but were not observed in our patients. The PRLTS5 patients are also characterized by progressive ataxia, axonal neuropathy, hyporeflexia, and abnormal eye movements as previously reported in Japanese patients (9), but these symptoms were also not observed in our cases.

Our primary clinical diagnosis in our patients, due to absence of neurological findings, pointed to either PRTLS2 or PRLTS4. However, after establishing the molecular diagnosis of CLPP pathogenicity, we concluded that p.Ile208M might have caused a milder PRLTS3 phenotype in our cases. On the other hand, it must be remembered that all the features may not always be prominent in PRLTS3 patients. For example, a previously reported Pakistani family, with splice donor-site mutation (c.270+A>G in CLPP), had only hearing loss with neither brain involvement nor any other associated abnormality (6). The clinical features of PRLTS3 are also age-dependent as described previously (7). Furthermore, we assume that hearing defect and ovarian dysgenesis without neurological findings might be a specific association with our mutation. Marlin et al (30) reviewed 34 cases from 15 families and reported hearing defect and ovarian dysgenesis without neurological findings. Our analyses encourage CLPP screening in such cases.

In conclusion, PRLTS is clinically diagnosed with the presence of primary ovarian failure in association with SNHL, and sometimes, with neuromuscular involvement. Clinical presentation is quite variable since the onset of all components may take time to appear. Gonadal insufficiency is not usual in boys and is noted only after pubertal age in girls. For these reasons, this syndrome should be suspected in patients presenting with unexplained neurologic findings and SNHL. The definite diagnosis of the type of PRLTS can be made only by molecular analyses since the clinical features may overlap. Patients with an identified mutation in the genes associated with PRLTS should be followed up in terms of clinical findings even if they are asymptomatic. Our patients were unique for having a novel mutation in CLPP gene, which leads to a mild form of PRLTS3 without any neurologic involvement. The genome-wide SNP microarray genotyping or array CGH followed by candidate gene sequencing may be used as a useful tool for PRTLS-causative variant identification in ethnically or geographically isolated familial cases.

## Acknowledgments

The authors, therefore, acknowledge with thanks DSR for technical and financial support. Noreen Karim was supported by an indigenous PhD fellowship from HEC, Islamabad, Pakistan.

## Ethics

Informed Consent: It was taken.

Peer-review: Externally peer-reviewed.

## Figures and Tables

**Table 1:**
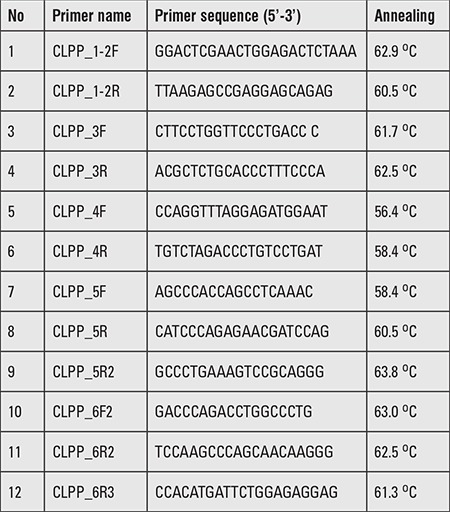
List of primers along with the annealing temperature used for polymerase chain reaction amplification of the six coding exons of CLPP gene
